# Portal Vein Embolization Using N-Butyl Cyanoacrylate-Glue: What Impact Does a Central Vascular Plug Have?

**DOI:** 10.1007/s00270-021-03014-w

**Published:** 2021-12-14

**Authors:** Ulrik Carling, Bård Røsok, Sigurd Berger, Åsmund Avdem Fretland, Eric Dorenberg

**Affiliations:** 1 Department of Radiology, Oslo University Hospital, Rikshospitalet, Postbox 4950 Nydalen, 0424 Oslo, Norway; 2 Department of Hepato-Pancreatic-Biliary Surgery, Oslo University Hospital, Oslo, Norway; 3 The Intervention Centre, Oslo University Hospital, Oslo, Norway

**Keywords:** Portal vein embolization, Colorectal liver metastases, Vascular plug, NBCA glue, Cone-beam CT

## Abstract

**Purpose:**

To examine if the addition of a central vascular plug (CVP) to portal vein embolization (PVE) with N-butyl cyanoacrylate-glue (NBCA) increases future liver remnant (FLR) growth.

**Material and Methods:**

This is a single-center retrospective study of 115 consecutive patients with colorectal liver metastases undergoing PVE in 2013–2019. All patients were embolized with NBCA as the main embolic agent. In 2017–2019 NBCA was combined with a CVP in the central part of the right portal vein. Growth of the FLR and standardized FLR (sFLR) including degree of hypertrophy (DH) and kinetic growth rate (KGR) were analyzed, as well as procedure data such as use of cone-beam CT (CBCT), dose area product (DAP), fluoroscopy time and contrast dose.

**Results:**

A total of 40 patients (35%) underwent PVE with a combination of CVP and NBCA. The DH was higher in these patients after 4 weeks, mean 13.6% (SD 7.8) vs. 10.5% (SD 6.4; *p* = 0.022), verified in multivariate analysis (coefficient 4.1, *p* = 0.015). A CVP did not significantly increase the resection rate (90% vs 82%, *p* = 0.4). Cone beam CT was used in 65 patients (57%). Use of CBCT did not affect FLR growth, and fluoroscopy time and contrast doses were not different in patients having a CBCT or not. Slightly lower DAP (median 3375 vs. 4499 cGy*cm^2^; *p* = 0.09) was seen in procedures where CBCT was used.

**Conclusion:**

A CVP in addition to NBCA embolization was associated with increased growth of the FLR compared to NBCA alone.

**Supplementary Information:**

The online version contains supplementary material available at 10.1007/s00270-021-03014-w.

## Introduction

Portal vein embolization (PVE) is an established method for improving patient outcomes following large liver resections for malignant liver tumors, such as metastases from colorectal carcinoma (CRLM) [1]. The goal of PVE is to stimulate growth of the intended future liver remnant (FLR) to reduce the risk for post hepatectomy liver failure (PHLF). Safe resection depends both on the size of the FLR relative to the size of the patient (the standardized FLR, sFLR), as well as underlying liver disease. Patients with CRLM are often treated with hepatotoxic chemotherapy and the minimum recommended volume threshold for safe surgery in these patients is a sFLR of 30% [2, 3].

Several methods have been described for PVE and different embolization materials have been used; often either glue (e.g. N-butyl cyanoacrylate; NBCA) or particles, sometimes combined with plugs or coils [1, 4]. It has been found that a combination of coils/vascular plugs and particles yields increased FLR growth compared to particles alone [5]. Several studies have indicated a benefit of using NBCA over particles [6–8] and this was recently confirmed in a randomized controlled trial including 60 patients [9]. Furthermore, a technique has been described where vascular plugs are combined with NBCA embolization to avoid non-target embolization of the FLR and reduce the re-canalization rate [7, 10], but there is no study demonstrating the actual benefit of this technique compared to embolization with NBCA alone.

The complex anatomy of the intrahepatic portal vein system can make PVE challenging and it is essential to avoid unintended embolization of the FLR. Standard digital subtraction angiography (DSA) has been the gold standard for procedural imaging. More recently, cone beam computed tomography (CBCT) has been introduced and used for liver embolization procedures as a supplement to DSA [11, 12]. CBCT has been shown to provide superior vessel and target visualization during transarterial embolization compared to DSA [13], however with a potential increase of the radiation dose to the patient [14]. It has not been determined what impact CBCT has on PVE.

During the past years, the PVE technique in our institution has been developed in that we have started to use central vascular plugs in combination with NBCA and increasingly used an intraprocedural contrast-enhanced CBCT for image guidance during the embolization. The aim of this study was to examine the outcomes of PVE to see if the addition of a central vascular plug to NBCA embolization increases FLR growth. We also examined the impact on FLR growth and patient radiation dose of intraprocedural contrast-enhanced CBCT.

## Material and Methods

In this single-center retrospective study data collection from digital journal and radiology systems was performed on patients that had undergone PVE between years 2013–2019. The data collection was approved by the data protection official with waiver of documentation of consent. To reduce possible confounding due to differences in tumor disease management and underlying liver parenchyma disease, only patients with colorectal liver metastases (CRLM) were included. For these patients a post PVE sFLR > 30% is aimed for before resection. Furthermore, due to the increasing use of both central plugs and CBCT over time, separate analyzes were made on patients without central plugs with regard to the impact of CBCT on FLR hypertrophy and patient radiation dose.

### Imaging

Volumetric data of the FLR, registered as part of the clinical routine, was obtained from the radiology information system. During the study period, routine liver volumetry was performed manually by trained staff at the radiology department on pre-PVE contrast-enhanced (portal venous phase) CT or MRI images. Post-PVE volumetry was performed on contrast-enhanced (portal venous phase) CT obtained 4 weeks after the PVE. Volumes were calculated by adding the area of multiple non-adjacent image slices, typically 3–4 slices apart, and then multiplying with the interval/distance between the measurements, typically 9–10 mm [15]. Large vessels and tumors in the FLR were not included in the volume. Any missing volumetry data was completed in the same manner by authors 1, 2 or 5. The sFLR, the degree of hypertrophy (DH) describing the sFLR change, and the kinetic growth rate (KGR) describing the sFLR change/week, were calculated as described by Shindoh et al. [16]. The post-PVE CT was retrospectively reviewed for any signs of glue in the FLR or other complications, and the distance between the portal vein bifurcation and the vascular plug was measured by authors 1 and 5. A central plug position was defined as the main right portal vein, and/or the main anterior or posterior sector stem in the right hemiliver.

### Embolization Technique

**Fig. 1:**
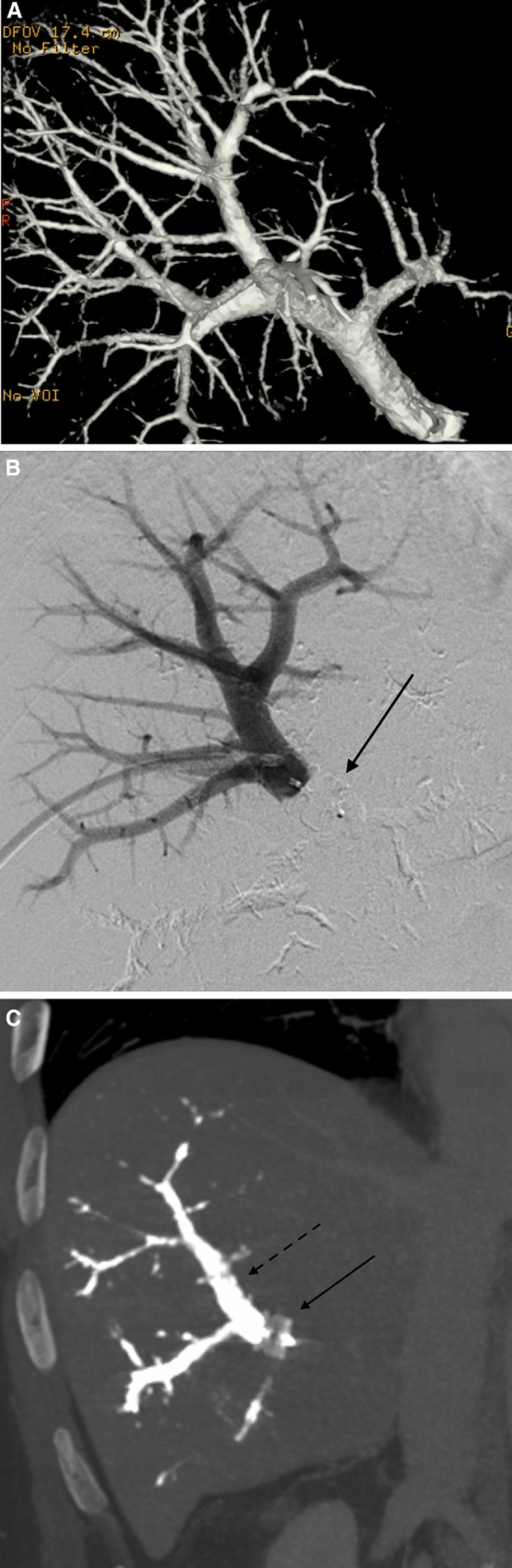
Portal vein embolization in a patient with colorectal liver metastases using a combination of glue and a central vascular plug guided by contrast-enhanced cone beam CT. **A**. Image of cone-beam CT 3D volume rendering which can be used as real-time overlay during fluoroscopy **B**. Digital subtraction angiography image after placement of a vascular plug (arrow) in the main right portal vein and into the main anterior sector stem after selective embolization of the posterior sector **C**. Maximum intensity projection image of contrast-enhanced CT 4 weeks after PVE showing the plug (black arrow) and glue cast (stapled black arrow) in the anterior sector

PVE was performed in local anesthesia and conscious sedation using intravenous administration of an opioid and benzodiazepine. Typically, two interventional radiologists collaborated during the procedures. The portal vein was accessed by an ultrasound-guided puncture of a peripheral ipsilateral portal vein branch (e.g. in the anterior part of the right hemiliver) using a 20G coaxial needle, microwire, and a 23 cm 4F radial vascular sheath (Cordis Corporation, Miami Lakes, USA). In cases where CBCT was used, this was performed with a 200° rotational angiography (GE Innova, GE Healthcare, Chicago, USA) using an angiographic catheter (e.g. 4F Universal Flush, Cordis Corporation, Miami Lakes, USA) in the main portal trunk. A 3:2 iodine contrast to saline mixture was injected at 5 ml/s, total 35 ml (2 s x-ray delay and 5 s rotation time). A 3D model of the contrast-enhanced portal vein tree was produced on the work-station (GE Healthcare, Chicago, USA) and was used as overlay during fluoroscopy (Fig. 1A). A reversed catheter (Simmons 1, Terumo Corporation, Tokyo, Japan) and a microcatheter (Cantata 2.8 F, Cook, Bloomington, USA) were used for selective catheterization of subsegmental portal vein branches. Embolization was made using a mixture of 1:4 NBCA glue (Histoacryl®, Braun, Melsungen, Germany) and lipiodol (Guerbet, Villepinte, France), and dextrose was used for flushing. In preparation for a planned extended right hepatectomy, embolization of segment 4 was performed through a microcatheter with either micro coils or NBCA. Successful embolization and patent portal vein flow in the FLR was verified by angiography. In some cases, access portal vein branch embolization was performed with a vascular plug (Amplatzer Vascular Plug IV, Abbott Medical, Plymouth, USA) and puncture tract embolization was performed with a gelatin sponge torpedo through the sheath. In the cases where a central vascular plug was used at the discretion of the operator, the 4F sheath was exchanged for a 6F sheath. Sizing of the plug (Amplatzer Vascular Plug II–AVP II, Abbott Medical, Plymouth, USA) was made according to the size of the portal vein on the CBCT, with 10–20% oversizing. The anatomy of the portal vein varies, but a separate left and right branch and a separate anterior and posterior sector branch from the right portal vein is most common [17]. With the sheath typically entering the anterior sector of the right hemiliver, the plug was placed in the main right and into the main anterior stem after selective NBCA embolization of the posterior sector of the right hemiliver (Fig. 1B). This was followed by embolization of the anterior sector distally to the plug (Fig. 1C) typically using a 4F glide catheter (Terumo, Tokyo, Japan) without selective catheterization of subsegmental branches. A glue-to-lipiodol rate of 1:8 was used to ensure distal distribution of the NBCA. No additional vascular plugs were used in patients with a central vascular plug, but puncture tract embolization often was done as described above. Technical success was defined as successful puncture and catheterization of the portal vein followed by NBCA embolization of non-FLR branches. Data on equipment, dose area product (DAP), fluoroscopy time, contrast volume, and periprocedural medication were recorded in the radiology systems at the end of the procedure. After the procedure, the patients were cared for in the surgical ward usually for one day. Complications were registered retrospectively according to the Cardiovascular and Interventional Radiological Society of Europe (CIRSE) classification [18].

In case of insufficient FLR growth (post-PVE sFLR < 30% and/or limited hypertrophy from pre-PVE volumetry) on the follow-up CT, the multidisciplinary team (MDT) decided either to refer the patient to re-PVE where obvious missed branches were seen on post-PVE CT, wait for additional growth or to perform a rescue ALPPS (Associating Liver Partition and Portal vein Ligation for Staged hepatectomy–ALPPS) in order to stimulate further growth [19, 20].

### Statistics

Statistical analyses were performed in IBM SPSS 26.0 (IBM Corp., Armonk, NY, USA). To compare two groups, t-test was used for normal distributed data and Mann–Whitney U-test for non-normal distributed continuous data, and 2 × 2 tables with Fisher’s exact test were used for categorical data. Following univariate analyses, independent variables with p-values less or equal to 0.2 were subsequently included in a multivariate linear regression analysis. Log-transformation of the dependent variable was used to if necessary to avoid skewed data. A *p*-value < 0.05 was considered as statistically significant.

## Results

**Fig. 2:**
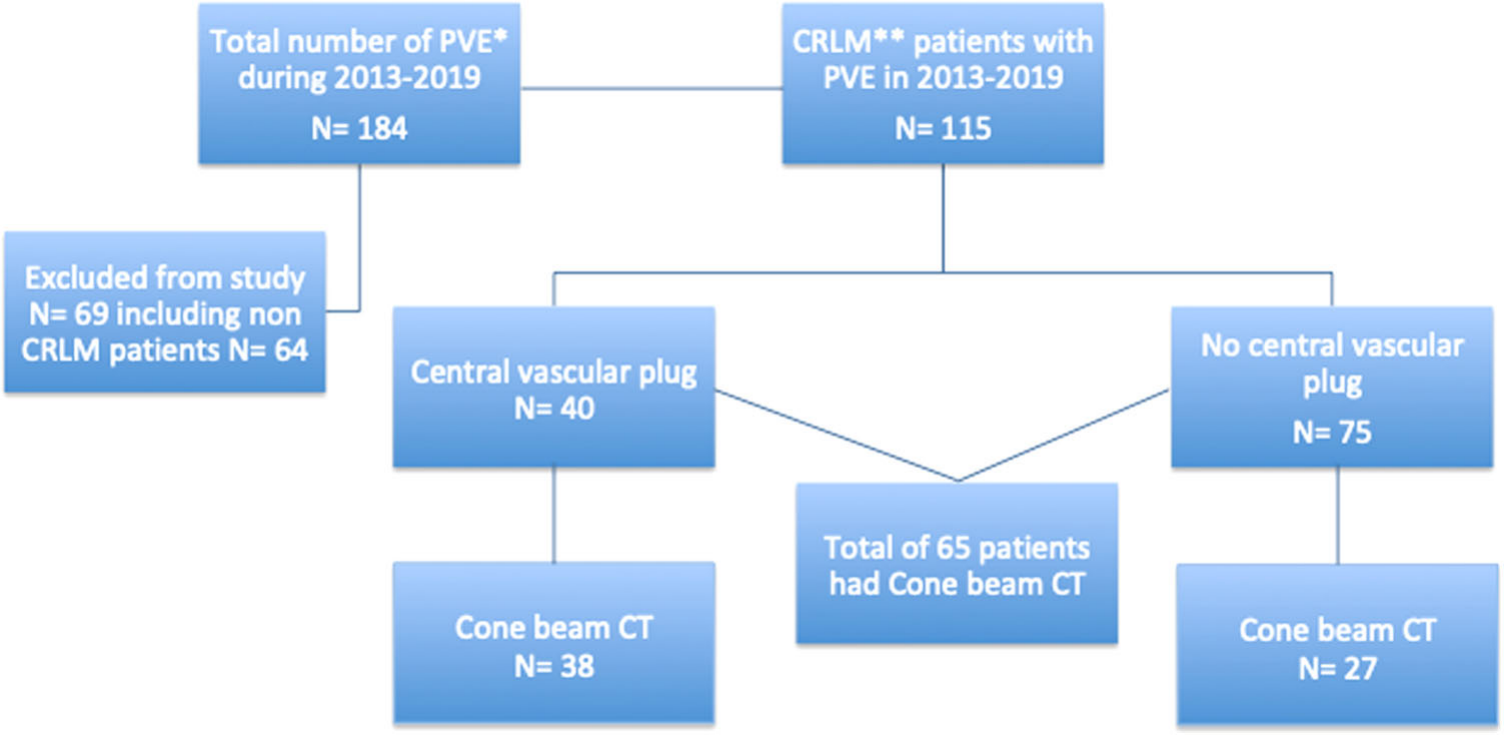
Flowchart of patient cohort. *Portal vein embolization, **Colo-rectal liver metastases

A total of 184 patients underwent PVE in our institution during the period of 2013–2019, and 69 patients were excluded from the analyses; 64 non-CRLM patients, two patients with sequential mini-ALPPS, one with a re-PVE from a different institution, and two patients with follow up and surgery at another institution. A flowchart of the patients can be seen in Fig. 2. Demographic and clinical data of the remaining 115 consecutive CRLM patients undergoing PVE can be seen in Table 1.

**Table 1:** Demographic and clinical data of 115 patients with colorectal liver metastases undergoing portal vein embolization

Characteristic	
Gender male (%)	81 (70.4)
Age years mean (SD)	66 (11.3)
Body mass index kg/m^2^ mean (SD)	25.7 (3.9)
Diabetes (%)	11 (9.5)
Cytostatic treatment (%)	110 (95.6)
Bilirubin median µmol/L (IQR)	8 (6–11)*
Largest tumor size median mm (IQR)	26.5 (13–40)
Tumor number median (IQR)	7 (3–11)

^*^No patients were icteric or had bile duct affection needing drainage

The technical success of PVE was 100% as embolization of the portal vein tree was made in all cases. The complications were mainly attributed to post embolization syndrome (PES) with fever and/or pain (Table 2). An overview of number of PVE procedures at the institution can be seen in Suppl. Table 1. A total of 40 (35%) patients had a central vascular plug (AVP II, size median 14 mm; range 8–18 mm) placed during the PVE. There were no significant differences between the groups with regard to the demographic data in Table 1, except that patients with a central plug had more tumors (10 vs 6; *p* < 0.01). The median distance from the bifurcation of the main portal vein to the plug was 10 mm (IQR 7–13 mm), describing the distance available for the surgeon to ligate the portal vein. One patient with a trifurcated portal vein (separate left, anterior and posterior right branches) had a 14 mm plug placed both in the anterior and posterior stem after separate punctures of both sectors. The combined use of a central plug and NBCA induced a significant increase in FLR growth compared to NBCA alone, with a DH of 13.6 vs. 10.5 (*p* = 0.02; Table 2). In multivariate analyses, a central plug was associated with both increased DH and KGR (Table 3). Furthermore, use of a central plug was associated with significantly shorter fluoroscopy time (Table 3). Data were missing for DAP, fluoroscopy time and contrast volume for one patient without a central plug, as well as contrast volume for one patient with a central plug.

**Table 2:** Pre portal vein embolization (PVE) data and outcomes comparing patients with a central plug versus no central plug

Result	Central plug (*N* = 40)	No central plug (*N* = 75)	*p*-value
Oxaliplatin cytostatic regime (%)	23 (57.5)	44 (58.7)	1.0
Pre PVE FLR cleaning^a^ (%)	18 (45)	34 (45)	0.36
Pre PVE MRI^b^ volumetry (%)	11 (27.5)	21 (28)	1.0
Cone-beam CT (%)	38 (95)	27 (36)	0.0001
Segment 4 embolization (%)	5 (12.5)	14 (18.7)	0.44
Pre FLR mean ml (SD)	435 (156)	396 (104)	0.11
Pre sFLR%^c^ (SD)	24.8 (6.1)	23.9 (5.4)	0.38
Pre PVE sFLR% < 20 (%)	8 (20)	23 (31)	0.27
Post FLR mean ml (SD)	653 (186)	562 (150)	0.005
Post sFLR% (SD)	38.5 (9.4)	34.4 (9.6)	0.03
Change FLR % (SD)	55.6 (32.3)	43.5 (24.4)	0.03
Weeks to CT control mean (SD)	4.5 (0.9)	4.4 (0.9)	0.58
Degree of hypertrophy % mean (SD)	13.6 (7.8)	10.5 (6.4)	0.02
Kinetic growth rate %/week mean (SD)	3.1 (1.9)	2.5 (1.6)	0.07
Dose area product cGy*cm^b^ median (IQR)	4607 (1793–7421)	4261 (2117–6405)	0.22
Fluoroscopy time minutes mean (SD)	38 (14.5)	50 (17.5)	0.00
Contrast ml mean (SD)	153 (54.7)	172 (67.8)	0.14
Opioids median mg (range)	10 (2.5–20)	7.5 (2.5–20)	0.04
Complication Grade*			
2	3	4	–
3	2	5	–
FLR glue^c^ (%)	1 (2.5)	8 (11)	0.16
Completed resection (%)	36 (90)	62 (82.7)	0.4
Weeks to surgery mean (SD)	9 (4.4)	8.4 (5.3)	0.52
Rescue ALPPS^d^, re-PVE** (%)	4 (10)	13 (17)	0.4
Severe PHLF^e^ (%)	2 (5)	7 (9.3)	0.5

^a^Resection or ablation in the future liver remnant (FLR) before portal vein embolization (PVE),

^b^Magnetic Resonance Imaging

^c^standardized FLR

^d^Any sign of glue in the FLR – very limited in all cases and no case of clinical relevance, ^e^Associating liver partition and portal vein ligation for staged hepatectomy

^e^Post hepatectomy liver failure level B (N = 5) or C (N = 4) as defined by International Study Group of Liver Surgery (ISGLS)[30]

^*^Complications as per Cardiovascular and Interventional Radiological Society of Europe.- 2; one case (non-plug group) of subcapsular hematoma seen in post PVE CT without symptoms, the rest was post embolization syndrome (PES) not needing any elevated care, 3; one (plug group) portal vein thrombus protruding into the main stem delaying surgery, one (plug group) pulmonary embolism (anticoagulation was withdrawn before PVE) and 5 cases (non-plug group) of PES needing in house care

^**^3 cases of re-PVE in no central plug group

**Table 3:** Regression analyses for degree of hypertrophy, kinetic growth rate, and fluoroscopy time in 115 patients with colorectal liver metastases undergoing portal vein embolization (PVE)

	Degree of hypertrophy (%)		Kinetic growth rate (%/week)		Fluoroscopy time (minutes)	
	Univariate	Multivariate	Univariate	Multivariate	Univariate	Multivariate
Gender^a^	3.65	2.14	1.0	0.62	0.38	NA
*p*-value	0.01	0.14	0.003	0.068	0.92	
Age (years)	0.04	NA	0,003	NA	− 0.12	NA
*p*-value	0.48		0.85		0.43	
BMI^b^	− 0.57	− 0.59	− 0.16	− 0.15	− 0.63	− 0.13
*p*-value	0.001	0.001	0.00	0.000	0.14	0.74
FLR clean^c^	− 0.38	NA	− 0.17	NA	− 1.00	NA
*p*-value	0.78		0.60		0.76	
CBCT^d^	1.72	− 0.79	0.33	NA	− 3.58	NA
*p*-value	0.19	0.60	0.31		0.28	
Central plug^e^	3.14	4.1	0.62	0,82	− 12.5	− 12.7
*p*-value	0.02	0.015	0.067	0,009	0.000	0.00
Segment 4^f^	− 0.72	NA	− 0.18	NA	16.3	15.5
*p*-value	0.68		0.69		0.000	0.000
sFLR% > 20^g^	2.81	1.2	0.72	0.39	− 4.43	NA
*p*-value	0.056	0.40	0.046	0.239	0.24	
Tumor size^h^	− 0.006	NA	0.002	NA	− 0.04	NA
*p*-value	0.79		0.78		0.54	
Tumor N^i^	0.15	0.07	0.03	NA	0.02	0.26
*p*-value	0.19	0.54	0.25		0.064	0.36

^a^Male vs female

^b^Body mass index kg/m^2^

^c^Surgery or ablation in the future liver remnant prior to PVE (no vs. yes)

^d^Periprocedural cone-beam CT (no vs. yes)

^e^Central vascular plug (no vs. yes)

^f^Embolization of segment 4 (no vs. yes)

^g^Standardized future liver remnant below or above 20%

^h^Size of largest tumor in mm

^i^Number of tumors

A total of 65 patients (57%) had an intraprocedural contrast-enhanced CBCT. The use of CBCT did not seem to affect the growth of the FLR (Table 3). As a CBCT was used in 95% of the cases where a central plug was placed, a separate analysis was made on the use of CBCT in patients without a central plug (and performed in the same angiosuite, *n* = 71; Table 4) to limit potential confounding. There was no impact on FLR growth. Furthermore, in the univariate analysis, CBCT was associated with a lower DAP. This was not confirmed in the multivariate analysis where only higher body mass index (BMI) was significantly associated with higher DAP (Table 5). There was no significant difference in BMI between patients with CBCT (mean 24.7; SD 2.5) or without CBCT (mean 25.3; SD 4.2, *p* = 0.48). The use of CBCT did not significantly influence fluoroscopy time or contrast dose.

**Table 4:** Outcomes with regard to cone-beam CT (CBCT) for patients in whom portal vein embolization (PVE) was performed without a central plug and in the same angiosuite (n = 71)

Result mean (SD)	CBCT (n = 26)	No CBCT (n = 45)	p-value
Degree of hypertrophy %	10.6 (8.1–13)	10.5 (8.6–12.4)	0.98
Kinetic growth rate %/week	2.5 (1.8–3.2)	2.5 (2.0–3.0)	0.96
Dose Area Product cGy*cm2 median (IQR)	3375 (1566–5184)	4499 (2294–6703)	0.09
Fluoroscopy time minutes mean (SD)	53 (20.2)	48 (16.0)	0.28
Contrast ml mean (SD)	166 (47.6)	174 (77.1)	0.62

**Table 5:** Regression analyses for dose area product (DAP; Gy*cm^2^)* in the 71 patients in Table 5

Variable	Univariate	p-value	Multivariate	*p*-value
Gender^a^	− 0.24	0.005	− 0.09	0.23
Age (years)	0.002	0.53	NA	
BMI^b^	0.05	0.000	0.04	0.000
CBCT^c^	− 0.12	0.13	− 0.1	0.14
Segment 4^d^	− 0.06	0.54	NA	
Number of tumors	− 0.002	0.82	NA	
Tumor size^e^	− 0.002	0.083	− 0.001	0.35

^*^Logarithmic transformation was used due to skewed data,

^a^Male vs. female

^b^Body mass index kg/m^2^

^c^Periprocedural cone-beam CT (no vs. yes)

^d^Embolization of segment 4 (no vs. yes)

^e^Size of largest tumor in mm

In total 98 (85%) patients completed resection after PVE. The use of a central vascular plug did not significantly increase the resection rate (90% vs 82%, *p* = 0.4), or affect the rate of PHLF (Table 2). In all 17 (14.8%) patients needed either rescue ALPPS (*n* = 14) or re-PVE (*n* = 3). The 3 patients (all in non-plug group) needing a re-PVE had slow hypertrophy and open right branches on post PVE CT. Rescue ALPPS in 14 patients (4 in the central plug group, 10 in non-plug group; *p* = 0.6) was performed median 7.7 weeks (range 6–15.6) after PVE. An sFLR below 20% (*n* = 26) was significantly associated with need for an additional volume expanding procedure (42.3% vs 8.3%, *p* = 0.0003). The 17 patients not having completed resection did so based on disease progression and not due to failure of FLR growth.

## Discussion

In this study, we found that PVE with a central vascular plug in combination with NBCA glue was associated with an increased growth of the future liver remnant compared to PVE with NBCA alone. Furthermore, increased BMI was associated with lower FLR growth, possibly due to increased liver steatosis in these patients [21], although this was not specifically analyzed. The method of using a central vascular plug together with NBCA has been described earlier in a series of 16 patients [10] and 45 patients [7], including a total of 24 patients with colorectal liver metastases. It was demonstrated the feasibility of using a plug in combination with NBCA as well as a clinical benefit compared to the combination of particles and coils [7]. However, none of these studies described a comparison with NBCA only. The plug allows for a complete NBCA cast in the portal vein tree distally to the plug while protecting from non-target embolization [10]. We did not quantify the potential differences in glue cast in the two cohorts, but when reviewing the post PVE CT we found slightly fewer cases with small glue fragments in the FLR in patients with a central plug. Furthermore, the central plug facilitates the occlusion of small branches often originating from the central part of the right portal vein, which also might add to a superior overall embolization effect. Using a central plug in addition to NBCA also has been described as a relatively fast procedure [7, 10]. The overall fluoroscopy time in our study was relatively high compared to earlier reports on PVE with NBCA probably due to extensive use of subsegmental catheterization with microcatheters in the present study [7, 9]. By using an additional plug we observed a shortening of the fluoroscopy time, likely due to reduced catheterization time as embolization behind the plug typically was performed without subsegmental catheterization with microcatheters.

The 1 cm distance between the plug and the take-off from the main portal vein allows some space for the surgeon to ligate the vessel [17], and resection rates were not significantly affected by the use of a central plug in this study. No patients were unable to be resected due to insufficient growth, but patients with an sFLR < 20% relatively more often needed an additional volume-expanding procedure prior to resection. This is in line with earlier reports [20], and it might be a useful cutoff for considering more invasive strategies upfront such as ALPPS or liver venous deprivation–simultaneous hepatic and portal vein embolization (double vein embolization) [22, 23].

The use of CBCT did not seem to affect the embolization result (FLR growth) neither in the multivariate analyzes of the whole cohort nor in sub-analyzes on the patients without a central plug. Some previous publications mention the use of CBCT in PVE [24–26], and also one study describes its use for accessing the portal vein tree [27]. Still, it seems to lack data on the use of CBCT during PVE, including the impact on radiation dose. Fluoroscopy time and contrast dose were comparable when using CBCT or not, likely as fluoroscopy is used during embolization also in CBCT-guided PVE. Regarding radiation dose, it has been described both an increase in DAP during transarterial embolization [14, 28] as well as a reduction in DAP when using CBCT compared to using DSA only [29]. In our study, DAP was lower when using CBCT and although this was associated with lower BMI, it seems that contrast-enhanced CBCT does not contribute to increased radiation or contrast dose to the patient during PVE. With the benefits of improved visualization, CBCT is now an integral part of the PVE procedure at our institution.

The retrospective method is a limitation of this study, and although PVE with NBCA glue has been performed since 2006 in our institution, there might have been a time bias as central plugs were used in the later part of the study period. However, this also facilitates the comparison since patient selection and management otherwise was consistent throughout the study period. Furthermore, not all pre- and post-procedural imaging was performed at the same institution; in fact, most of the pre-PVE imaging was performed outside our institution at local hospitals which allowed for different imaging protocols and thereby heterogeneous image quality. Specifically, both MRI and CT were used interchangeably for pre-PVE volumetries. This practice, as well as the method for liver volumetry, was the same throughout the study period, thereby reducing the risk for these limitations to confound the findings of the study.

In conclusion, the use of a central vascular plug in addition to NBCA glue embolization was associated with increased growth of the FLR compared to NBCA alone in this study. Contrast-enhanced CBCT can be used during the procedure without increasing the overall radiation and contrast doses to the patients. Prospective controlled studies are needed to verify these findings.

## Supplementary Information

Below is the link to the electronic supplementary material.

Supplementary file1 (DOCX 12 kb)
