# Supplementary material for: Portal Vein Embolization Using N-Butyl Cyanoacrylate-Glue: What Impact Does a Central Vascular Plug Have?
Source: Cardiovasc Intervent Radiol. 2021 Dec 14;45(4):450–8. doi: 10.1007/s00270-021-03014-w (PMC8940786; doi:10.1007/s00270-021-03014-w)
Supplement: Supplementary file 1 — Supplementary file1 (DOCX 12 kb) [file 270_2021_3014_MOESM1_ESM.docx]

Supplementary Table 1 Number of procedures - portal vein embolization for patients with colorectal liver metastases 2013-2019

| Year | 2013 | 2014 | 2015 | 2016 | 2017 | 2018 | 2019 | Total |
| --- | --- | --- | --- | --- | --- | --- | --- | --- |
| Total (N) | 12 | 11 | 12 | 24 | 10 | 23 | 23 | 115 |
| CBCT^1^ (N) | 1 | 2 | 1 | 9 | 7 | 23 | 22 | 65 |
| Central plug^2^ (N) | 0 | 0 | 0 | 1 | 5 | 14 | 20 | 40 |

^1^Use of intraprocedural cone beam CT, ^2^use of a central vascular plug in the right portal vein in addition to glue embolization
